# Serum tumor markers level and their predictive values for solid and micropapillary components in lung adenocarcinoma

**DOI:** 10.1002/cam4.4645

**Published:** 2022-03-14

**Authors:** Zhihua Li, Weibing Wu, Xianglong Pan, Fang Li, Quan Zhu, Zhicheng He, Liang Chen

**Affiliations:** ^1^ Department of Thoracic Surgery Jiangsu Province Hospital, The First Affiliated Hospital of Nanjing Medical University Nanjing China

**Keywords:** histological subtypes, lung adenocarcinoma, predictive models, serum tumor markers, solid and micropapillary components

## Abstract

**Background:**

This study aims to reveal the serum tumor marker (STM) levels in lung adenocarcinoma (LUAD) histological subtypes and evaluate their values in predicting the solid and micropapillary components (SMC).

**Methods:**

We retrospectively analyzed 3100 invasive LUAD patients between January 2017 and December 2020. Associations between preoperative STMs (CEA, CYFRA21‐1, CA199, CA724, NSE, AFP) and LUAD subtypes were evaluated. Multivariate regression analyses were used to determine the independent predictors. Predictive models for SMC were constructed and AUC (area under the curve) was calculated.

**Results:**

CEA and CYFRA21‐1 levels differed across the LUAD histological subtypes, with the SPA (solid‐predominant adenocarcinoma) having the highest level and the LPA (lepidic‐predominant adenocarcinoma) harboring the lowest level (*p* <0.001). Tumors with SMC also had higher CEA and CYFRA21‐1 levels than those absence of SMC. Gender, tumor size, CEA, Ki‐67, EGFR mutation (solid components only), and tumor differentiation were significantly independently associated with the containing of SMC. Patients were split into two data sets (training set: 2017–2019 and validation set: 2020). The model with gender and tumor size yielded an AUC of 0.723 (training set) and 0.704 (validation set) for the solid component. Combination of CEA, gender, and tumor size led to a significant increase in the predictive accuracy (training set: 0.771, *p* = 0.009; validation set: 0.747, *p* = 0.034). The AUC of the model for micropapillary component with only gender and tumor size was 0.699 and 0.711 in the training set and validation set, respectively. Integration of CEA with gender and tumor size significantly improved the predictive performance with an AUC of 0.746 (training set, *p* = 0.045) and 0.753 (validation set, *p* <0.001).

**Conclusion:**

Serum CEA and CYFRA21‐1 varied considerably according to LUAD histological subtypes. The combination of serum CEA and other factors showed prominent values in predicting the SMC.

## Graphical abstract

Lung adenocarcinoma with solid or micropapillary components had higher levels of serum CEA and CYFRA21‐1 than those absence of solid and micropapillary components.Combination of serum CEA and other clinicopathological factors showed prominent values in predicting the solid and micropapillary components in lung adenocarcinoma.

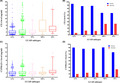

## 1 INTRODUCTION

Lung cancer is one of the most diagnosed and death‐related cancers in the world. The prognosis of lung cancer is poor and the overall 5‐year survival rate remains less than 30% in most countries.^1^, ^2^ Lung adenocarcinoma (LUAD) is the main histological subtype of lung cancer and accounts for about half of all lung cancer cases. As the great development in histopathology and molecular biology, LUAD was further classified into five histological subtypes: lepidic (LPA), acinar (APA), papillary (PPA), micropapillary (MPA), and solid‐predominant adenocarcinoma (SPA). Compared with LPA/APA/PPA subtypes, MPA and SPA subtypes had a higher risk of lymph node (LN) metastasis, tumor recurrence, and therefore an inferior survival.^3^, ^4^, ^5^ Therefore, the preoperative identification of solid and micropapillary components (SMC) is crucial for surgery decisions and prognostic predictions.^3^, ^6^

Over the past few years, the radiomics features of LUAD subtypes were depicted and models for predicting the subtype components, especially the SMC, were initially constructed.^7^, ^8^, ^9^, ^10^, ^11^ Most of these models showed a medium predictive efficiency with an accuracy of about 0.75. Recently, He et al. constructed four models based on five radiomics features, which achieved AUC (area under the curve) ranging from 0.69 to 0.75.^11^ Except for radiomics, researchers are trying to identify novel biomarkers to classify the histological subtypes of LUAD. For example, Zhao and colleagues detected the expression of adhesion and apoptosis molecules using antibody arrays in LUAD tumor tissues with and without micropapillary or solid components and found that insulin‐like growth factor‐binding protein 2 and P‐cadherin could identify micropapillary or solid components in LUAD with an accuracy of 80.9% in a short processing time.^12^

Traditional serum tumor markers (STMs), such as CEA (carcinoembryonic antigen), CA199 (carbohydrate antigen199), and NSE (neuron‐specific enolase), are widely used for the early diagnosis and classification of lung cancer.^13^, ^14^, ^15^ For example, TK1 (thymidine kinase 1) integration with CEA, CYFRA21‐1, and NSE achieved a diagnostic accuracy of 0.946 for benign and malignant lung tumors.^16^ The combination of serum levels of xanthine, SAM (S‐adenosyl methionine), CEA, SCC (squamous cell carcinoma antigen), and NSE showed a remarkable prediction accuracy (more than 90%) for the classification of LUAD, squamous cell carcinoma, and small cell carcinoma.^17^ However, whether the expression of STMs differed between histological subtypes of LUAD and whether these markers could be used to predict the SMC in LUAD largely remained unclear.

To the best of our knowledge, few studies have described the level of STMs in LUAD histological subtypes.^18^, ^19^ In 2015, Lu et al. found that the CEA level in patients with LPA was significantly lower than that in samples without LPA.^18^ Not long after that, Wang et al. found that SPA had a higher CEA level than other histological subtypes.^19^ However, these studies just analyzed the CEA level in histological subtypes of LUAD, other common biomarkers, such as CA199, NSE, CA199, and so on, were not explored. Besides, the sample size was limited, especially patients with SPA and MPA. Most importantly, they did not evaluate the value of STMs in predicting the SMC.

In the current study, we systematically evaluated the associations between six STMs (CEA, CA199, CA724, NSE, AFP, CYFRA21‐1) and LUAD histological subtypes, as well as other clinicopathological factors, by retrospectively analyzing the characteristics of 3100 invasive LUAD samples from January 2017 to December 2020 in our department. Further, predictive models for the SMC were constructed based on STMs and other clinicopathological characteristics. This study will provide a deeper insight into the levels of STMs according to histological subtypes of LUAD and an applied tool to predict the SMC in LUAD.

## 2 MATERIALS AND METHODS

### 2.1 Study subjects

We retrospectively screened lung cancer patients who underwent surgical treatment in our department between January 2017 and December 2020. Patients who met the following criteria were initially included in this study: (1) primary lung cancer; (2) histopathological confirmed invasive adenocarcinoma with precise subtype classification: LPA, APA, PPA, MPA, or SPA; (3) single invasive tumor nodule; (4) STMs detection within 1 month before the surgery. Further, patients who (1) received preoperative treatments, including chemotherapy, radiotherapy, target therapy, and immune therapy, and (2) had a history of other malignant tumors in 5 years were excluded.

### 2.2 Information extraction of study subjects

In this study, basic information and clinicopathological characteristics of study subjects were extracted from the medical records in our department. All these data were cleared up by one researcher and reviewed by another investigator. STMs were measured by electrochemiluminescent assay. The normal upper limit for CEA, AFP, CA199, CA724, CYFRA21‐1, and NSE was 4.7, 20.0 ng/ml, 39.0, 6.9 U/ml, 3.3, and 16.3 ng/ml, respectively. Tumors with the micropapillary or solid components <5% of the entire tumor were defined as absent.

### 2.3 Statistical analysis

Student's *t* test, one‐way ANOVA, or Kruskal test was used for the comparison of continuous variables. The chi‐square χ^2^ test or Fisher's exact test was adopted for categorical variables. STMs and Ki‐67 levels were shown as the median and interquartile range (IQR). Correlation between STMs and Ki‐67 was evaluated using Pearson correlation. The impacts of various factors on STM levels were estimated by a generalized linear model. Independent factors identified in multivariate regression analyses were reserved to predict the SMCs in LUAD. We depicted the ROC (receiver operating characteristic curve) using the “pROC” package. All the analyses were performed based on R (3.6.0), and *p* < 0.05 was considered statistically significant.

## 3 RESULTS

### 3.1 Characteristics of study subjects

As summarized in Table S1, this study enrolled a total of 3100 invasive LUAD patients. There were 1227 males and 1873 females with a mean age of 59.22 years old. The mean tumor size was 17.11 ± 8.69 mm. Specifically, there were 729 (23.52%), 1637 (52.81%), 546 (17.61%), and 188 (6.06%) patients with a tumor size ≤10, 10–20, 20–30 mm, and more than 30 mm, respectively. The number of patients with LPA, APA, PPA, SPA, and MPA was 931 (30.03%), 1933 (62.35%), 135 (4.35%), 80 (2.58%), and 21 (0.68%), respectively.

### 3.2 STM levels in histological subtypes of LUAD

**FIGURE 1:**
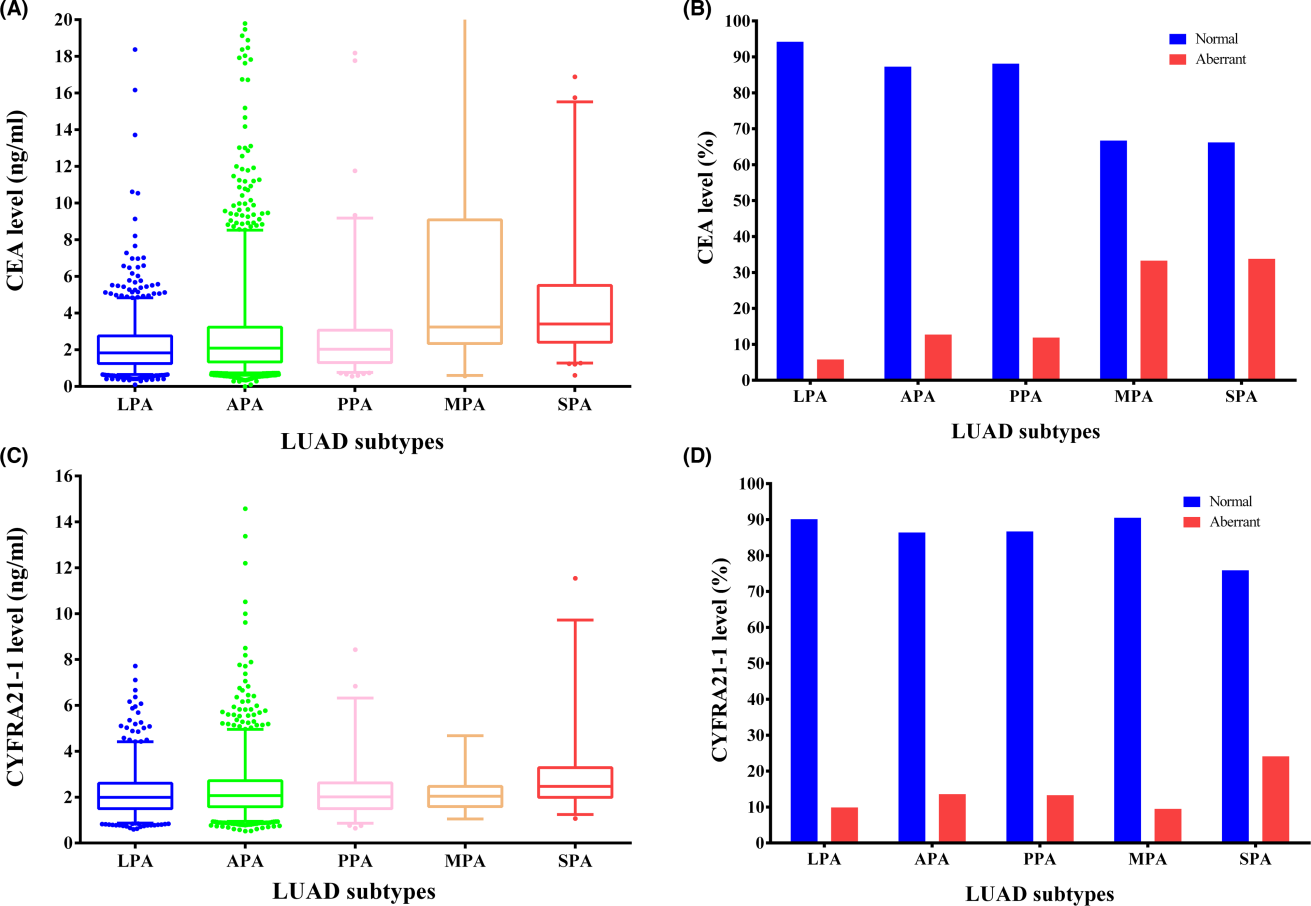
Serum CEA and CYFRA21‐1 expression levels in histological subtypes of lung adenocarcinoma. (A) Preoperative serum CEA differed across histological subtypes of LUAD; (B) MPA and SPA patients had higher percentages of abnormal CEA levels than LPA/APA/PPA; (C) CYFRA21‐1 levels varied according to LUAD subtypes; (D) Patients with SPA had a higher abnormal level of CYFRA21‐1 than those with other histological subtypes

As shown in Table 1 and Figure 1A,B, among these STMs, CEA and CYFRA21‐1 levels varied considerably according to the predominant histological subtypes of LUAD. The SPA subtype had the highest CEA level (3.41 [2.42–5.50]), followed by MPA (3.24 [2.42–5.69]), APA (2.09 [1.34–3.23]), PPA (2.03 [1.30–3.00]), and LPA (1.83 [1.25–2.76]) (*p* <0.001). Consistently, 33.8% of SPA patients had an abnormal CEA expression, while the proportion was 33.3% in MPA, 12.7% in APA, 11.9% in PPA, and 5.8% in LPA (*p* < 0.001). Similarly, the level of CYFRA21‐1 in the SPA subtype was significantly higher than that in other histological subtypes (*p* < 0.001). Only 9.9% of LPA subjects harbored an abnormal CYFRA21‐1 expression, while the proportion was 24.1% in patients with SPA.

**TABLE 1:** Comparison of baseline and STMs levels between histological subtypes of LUAD

Characteristics	LPA	APA	PPA	SPA	MPA	*p*
	*n* = 931	*n* = 1933	*n* = 135	*n* = 80	*n* = 21	
Gender = female	580 (62.3%)	1183 (61.2%)	78 (57.8%)	22 (27.5%)	10 (47.6%)	**<0.001**
Age	58.22 ± 10.46	59.67 ± 10.81	58.01 ± 9.40	61.61 ± 8.98	61.71 ± 7.63	**0.001**
Tumor size (cm)	1.37 ± 0.62	1.82 ± 0.87	1.84 ± 0.99	2.67 ± 1.24	2.71 ± 1.27	**<0.001**
Ki‐67	8.00 (5.00–10.00)	10.00 (5.00–25.00)	15.00 (8.00–25.00)	50.00 (30.00–75.00)	30.00 (15.00–50.00)	**<0.001**
AFP	2.67 (1.90–3.74)	2.78 (1.99–3.97)	3.02 (2.01–4.17)	2.31 (1.78–3.52)	3.09 (2.38–4.36)	0.050
CEA	1.83 (1.25–2.76)	2.09 (1.34–3.23)	2.03 (1.30–3.00)	3.41 (2.42–5.50)	3.24 (2.42–5.69)	**<0.001**
CA199	10.00 (6.71–15.35)	10.16 (6.70–15.68)	10.52 (7.01–14.67)	11.61 (5.68–21.73)	13.26 (6.05–28.07)	0.580
CA724	1.72 (1.11–3.63)	1.75 (1.07–3.84)	1.93 (1.09–3.82)	2.02 (1.21–5.46)	7.06 (1.63–11.76)	0.059
CYFRA21‐1	1.99 (1.50–2.60)	2.07 (1.58–2.72)	2.01 (1.52–2.61)	2.47 (1.99–3.27)	2.04 (1.60–2.43)	**<0.001**
NSE	16.92 (14.44–20.32)	16.51 (14.11–19.84)	16.77 (14.13–20.63)	17.67 (14.98–21.87)	16.86 (15.00–21.73)	0.063
AFP_positive(%)^a^	2 (0.2%)	2 (0.1%)	0 (0.0)	0 (0.0)	1 (4.8%)	**<0.001**
CEA_ positive(%)^a^	54 (5.8%)	246 (12.7%)	16 (11.9%)	27 (33.8%)	7 (33.3%)	**<0.001**
CA199_positive(%)^a^	13 (1.4%)	32 (1.7%)	3 (2.2%)	6 (7.5%)	1 (4.8%)	**0.002**
CA724_positive(%)^a^	95 (10.2%)	206 (10.7%)	17 (12.6%)	10 (12.7%)	9 (42.9%)	**<0.001**
CYFRA21‐1_positive(%)^a^	92 (9.9%)	262 (13.6%)	18 (13.3%)	19 (24.1%)	2 (9.5%)	**0.002**
NSE_positive(%)^a^	526 (56.5%)	1008 (52.2%)	69 (51.1%)	44 (55.7%)	12 (57.1%)	0.263

Abbreviations: APA, acinar‐predominant adenocarcinoma; LPA, lepidic‐predominant adenocarcinoma; MPA, micropapillary‐predominant adenocarcinoma; PPA, papillary‐predominant adenocarcinoma; SPA, solid‐predominant adenocarcinoma.

^a^ Patients with abnormal levels of STMs.

*P* value of <0.05 was marked in bold.

To confirm the differential CEA and CYFRA21‐1 levels between histological subtypes in LUAD, we further analyzed the levels of STMs in samples with and without solid or micropapillary components (Table 2). There were 397 and 384 patients harboring the solid and micropapillary components, respectively. Consistent with the above findings, patients with solid components had higher CEA and CYFRA21‐1 levels than those without solid components (*p* < 0.001). The percentages of patients with aberrant CEA and CYFRA21‐1 levels were 30.7% and 18.4% in patients with solid components, while only 8.4% and 11.8% in those without solid components (*p* < 0.001). In accordance, patients harboring micropapillary components had higher CEA (*p* < 0.001) and CYFRA21‐1 (*p* = 0.008) levels than that absence of micropapillary content. There were 27.9% and 17.4% of patients with micropapillary components had abnormal CEA and CYFRA21‐1 levels, which were much higher than 8.9% (*p* < 0.001) and 12.0% (*p* = 0.004) in patients without micropapillary components.

**TABLE 2:** Characteristics of LUAD subjects with/without solid or micropapillary components

Characteristics	Solid components		*p*	Micropapillary components		*p*
	Without (*n* = 2703)	With (n = 397)		Without (*n* = 2716)	With (*n* = 384)	
Gender = female (%)	1708 (63.2%)	165(41.6%)	**<0.001**	1676 (61.7%)	197 (51.3%)	**<0.001**
Age	59.06 ± 10.63	60.36 ± 10.43	**0.022**	59.14 ± 10.61	59.78 ± 10.63	0.273
Tumor size(cm)	1.61 ± 0.79	2.39 ± 1.07	**<0.001**	1.62 ± 0.79	2.38 ± 1.08	**<0.001**
Ki‐67	10.00 (5.00–15.00)	40.00 (25.00–60.00)	**<0.001**	10.00 (5.00–20.00)	25.00 (15.00–40.00)	**<0.001**
AFP	2.75 (1.96–3.89)	2.72(1.97–3.96)	0.965	2.73 (1.95–3.87)	2.83 (1.99–4.17)	0.287
CEA	1.93 (1.28–2.95)	2.94 (1.90–5.45)	**<0.001**	1.97 (1.29–2.99)	2.72 (1.54–5.29)	**<0.001**
CA199	10.11 (6.70–15.55)	10.61 (6.64–17.08)	0.487	10.11 (6.68–15.68)	10.37 (7.16–16.02)	0.381
CA724	1.77 (1.09–3.81)	1.74 (1.06–4.20)	0.606	1.75 (1.08–3.84)	1.75 (1.10–3.94)	0.577
CYFRA21‐1	2.04 (1.54–2.66)	2.18 (1.68–2.95)	**<0.001**	2.05 (1.55–2.67)	2.16 (1.63–2.79)	**0.008**
NSE	16.68 (14.27–20.05)	16.52 (13.94–20.27)	0.511	16.66 (14.25–20.06)	16.75 (14.15–20.37)	0.986
AFP_positive(%)	4 (0.1%)	1 (0.3%)	1.000	3(0.1%)	2(0.5%)	0.232
CEA_positive(%)	228 (8.4%)	122 (30.7%)	**<0.001**	243(8.9%)	107(27.9%)	**<0.001**
CA199_positive(%)	44 (1.6%)	11 (2.8%)	0.159	42(1.6%)	13(3.4%)	**0.019**
CA724_positive(%)	282 (10.5%)	55 (13.9%)	**0.048**	293(10.8%)	44(11.5%)	0.773
CYFRA21‐1_positive(%)	320 (11.8%)	73 (18.4%)	**<0.001**	326(12.0%)	67(17.4%)	**0.004**
NSE_positive(%)	1452 (53.8%)	207 (52.3%)	0.617	1454(53.6%)	205(53.4%)	0.982

*P* value of <0.05 was marked in bold.

### 3.3 STM levels according to gender, age, tumor sizes, LN status, and tumor differentiation grades

We further analyzed the levels of STMs in subgroup populations based on gender, age, tumor size, lymph node metastasis, and tumor differentiation grades. As shown in Table S2, males had higher CEA, CYFRA21‐1, and NSE levels than females (*p* < 0.001). Compared with younger patients (<60 years), the older had higher levels of CEA (2.39 [1.58–3.55] vs. 1.67 [1.11–2.56]) and CYFRA21‐1 (2.22 [1.72–2.91] vs. 1.88 [1.40–2.44]) (*p* < 0.001). The proportion of abnormal CEA and CYFRA21‐1 levels in older patients were 14.8% and 16.6%, which were much higher than that in younger patients (7.5% and 8.4%, *p* < 0.001, Table S3).

The levels of STMs according to tumor size, LN metastasis, and tumor differentiation were further estimated. Among these markers, CEA, CA199, and CYFRA21‐1 levels significantly increased with the increase in tumor size (*p* < 0.05), whereas AFP, CA724, and NSE showed no significant differential expression levels in patients with different tumor sizes (*p* >0.05). The median level of CEA increased from 1.59 ng/ml in tumors ≤10 mm to 1.95 ng/ml (10–20 mm), 2.77 ng/ml (20–30 mm), and 3.39 in tumors >30 mm in diameter (Figure S1 and Table S4, *p* < 0.001). Consistently, abnormal rate of CEA was 3.8%, 7.9%, 22.7%, and 36.2% in tumors ≤10, 10–20, 20–30, and >30 mm, respectively (*p* < 0.001). Similarly, the expression levels of CEA and CYFRA21‐1 in samples with LN metastasis (N+) were significantly higher than that in LN‐negative (N0) subjects (*p* < 0.001, Table S5). The abnormal rates of CEA and CYFRA21‐1 were 41.9% and 25.7% in the N+ arm, which was also much higher than that in N0 patients (8.9% and 11.7%, *p* < 0.001, Table S5), respectively. Besides, CEA and CYFRA21‐1 levels differed in tumors with different differentiation grades (Table S6). Tumors with a lower differentiation grade (ΙΙΙ) had higher CEA and CYFRA21‐1 levels than those with middle (ΙΙ) or high (Ι) differentiation grades (*p* < 0.001).

### 3.4 Univariate and multivariate regression analyses of CEA and CYFRA21‐1 levels

Given the differential levels of CEA and CYFRA21‐1 in histological subtypes and other subgroups, we further evaluated the associations between these clinicopathological factors, CEA and CYFRA21‐1 based on GLM regression models. As presented in Table S7, age, gender, tumor size, histological subtypes, EGFR mutation, and tumor differentiation grades were significantly associated with the abnormal level of CEA in both univariate and multivariate regression analyses (*p* < 0.05). In brief, older patients, male patients, larger tumor size, solid, or micropapillary components were associated with a higher probability of abnormal CEA level, whereas female patients, EGFR mutation, and high grade of tumor differentiation were associated with a lower risk of aberrant level of CEA. These results suggested that the impact of histological subtypes on CEA levels was not dependent on tumor size, age, and gender.

In contrast with CEA, gender, EGFR mutation and tumor differentiation showed no significant association with the abnormal level of CYFRA21‐1 in multivariate analysis (*p* >0.05). Older patients and patients with larger tumor sizes had a higher risk of abnormal CYFRA21‐1 levels (*p* < 0.001). Compared to the LPA subtype, SPA had a higher probability of CYFRA21‐1 abnormal expression (OR = 1.86, 95% CI: 1.02–3.39, *p* = 0.043, Table S7).

### 3.5 STMs levels and Ki‐67 expression in LUAD tumor tissues

In our previous study, we found that Ki‐67 expression differed across LUAD histological subtypes.^20^ Most importantly, we revealed that Ki‐67 expression level and tumor size could account for the survival differences between LUAD histological subtypes, at least partially. Given the vital role of Ki‐67 in the prognosis of LUAD, we further explored the correlation between STM levels and Ki‐67 expression. Overall, the CEA and CYFRA2‐1 showed consistent expression distributions with Ki‐67 in LUAD patients. As shown in Figure S2, the levels of CEA (*r* = 0.349, *p* < 0.001) and CYFRA21‐1 (*r* = 0.142, *p* < 0.001) in preoperative serum were significantly correlated with the expression of Ki‐67 in LUAD tissues. AFP, CA199, CA724, and NSE levels showed no significant correlation with Ki‐67 expression (*p* > 0.05).

### 3.6 Combination of STMs and other clinicopathological factors to predict the SMC in LUAD

The univariate regression analysis showed that age, gender, tumor size, CEA, CYFRA21‐1, Ki‐67, tumor differentiation, and EGFR mutation were significantly associated with the solid components (Table 3). However, age and CYFRA21‐1 were no longer significant in the multivariate analysis. Tumor size, gender, CEA, CYFRA211, Ki‐67, and tumor differentiation showed significant associations with the micropapillary components. After adjusting for tumor size and other variables identified in the univariate analysis, the CYFRA21‐1 level was not significantly associated with the micropapillary components at all (Table 3). Therefore, these promising factors identified in the multivariate analyses were reserved for the construction of predictive models.

**TABLE 3:** Univariate and multivariate regression analyses of solid and micropapillary components

Characteristics	Solid component				Micropapillary component			
	Univariate		Multivariate		Univariate		Multivariate	
	OR	*p*	OR	*p*	OR	*p*	OR	*p*
Age	1.01 (1.00–1.02)	**0.023**	1.00 (0.99–1.01)	0.540	1.01 (1.00–1.02)	0.273	/	/
Gender (female)	0.41 (0.33–0.51)	**<0.001**	0.46 (0.37–0.58)	**<0.001**	0.65 (0.53–0.81)	**<0.001**	0.78 (0.62–0.98)	**0.034**
Tumor size	2.27 (2.04–2.54)	**<0.001**	2.19 (1.94–2.47)	**<0.001**	2.21 (1.98–2.47)	**<0.001**	2.11 (1.87–2.37)	**<0.001**
CEA	1.04 (1.03–1.06)	**<0.001**	1.02 (1.00–1.04)	**0.009**	1.06 (1.04–1.08)	**<0.001**	1.03 (1.01–1.04)	**<0.001**
CYFRA21‐1	1.16 (1.08–1.25)	**<0.001**	0.99 (0.91–1.07)	0.745	1.14(1.06–1.23)	**<0.001**	0.96 (0.88–1.04)	0.353
AFP	1.01 (0.99–1.03)	0.185	/	/	1.02 (1.00–1.04)	0.108	/	/
CA199	1.00 (1.00–1.01)	0.552	/	/	1.00 (1.00–1.01)	0.060	/	/
CA724	1.00 (0.99–1.01)	0.941	/	/	1.00 (1.00–1.01)	0.380	/	/
NSE	1.00 (0.98–1.02)	0.796	/	/	1.00 (0.98–1.02)	0.953	/	/
Ki‐67	1.09 (1.08–1.11)	**<0.001**	1.04 (1.03–1.06)	**<0.001**	1.03 (1.03–1.04)	**<0.001**	1.03 (1.02–1.03)	**<0.001**
Differentiation		**<0.001**		**<0.001**		**<0.001**		**<0.001**
ΙΙΙ	Ref.	/	Ref.	/	Ref.	/	Ref.	/
ΙΙ	0.02 (0.01–0.04)	**<0.001**	0.03 (0.01–0.07)	**<0.001**	0.08 (0.05–0.11)	**<0.001**	0.08 (0.05–0.12)	**<0.001**
Ι	0 (0–0.01)	<0.001	0 (0–Inf)	0.978	0 (0–0.01)	**<0.001**	0 (0–0.02)	**<0.001**
EGFR (mutation)	0.46 (0.34–0.62)	**<0.001**	0.55 (0.39–0.78)	**<0.001**	0.74 (0.53–1.03)	0.070	/	/

*P* value of <0.05 was marked in bold.

**FIGURE 2:**
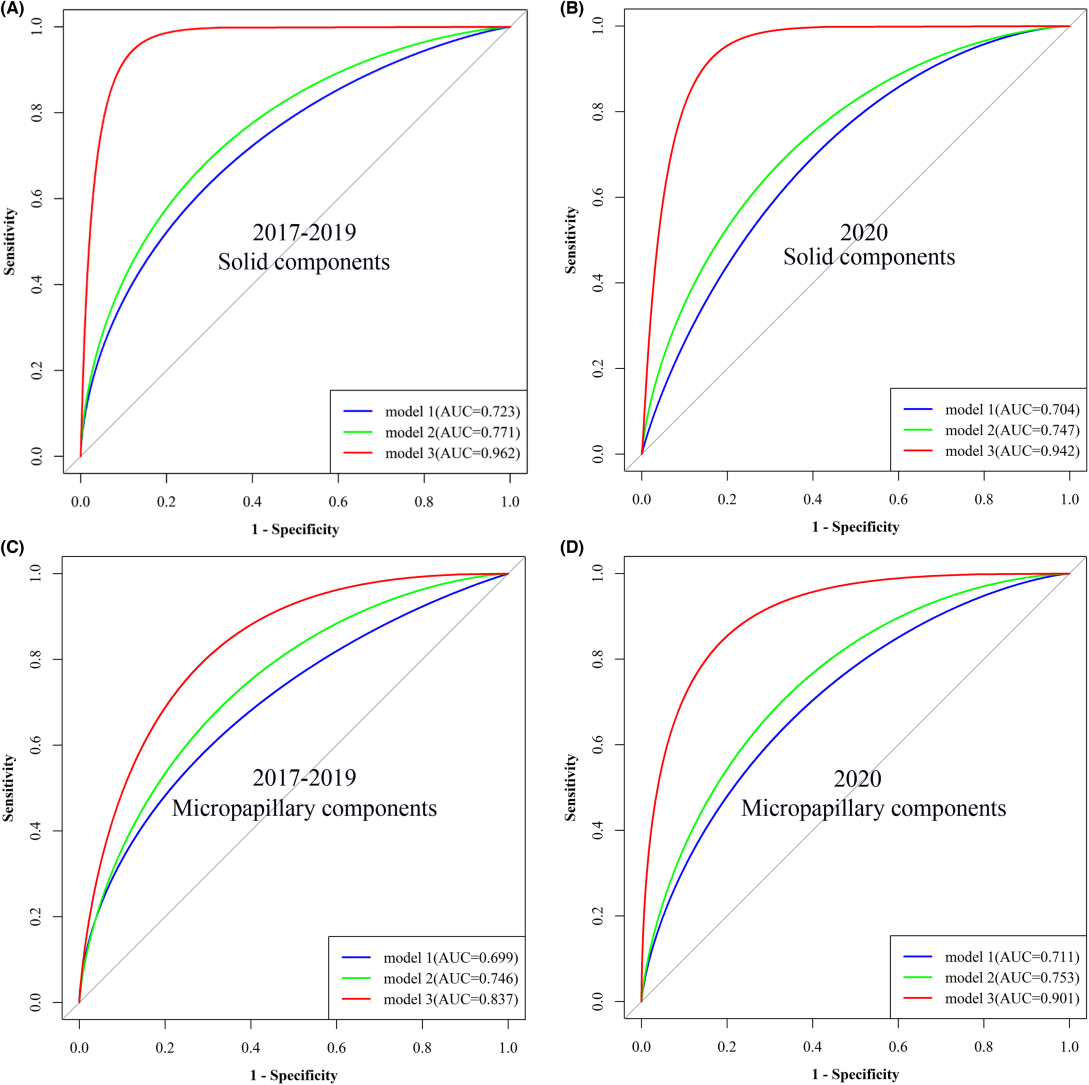
Predictive models for the solid and micropapillary components in LUAD samples. (A and B) Predictive models for solid components in the training set (A, 2017–2019) and in the validation set (B, 2020). Model 1: tumor size + gender; Model 2: Molde 1 + CEA; Model 3: Model 2 + Ki‐67 + EGFR mutation + tumor differentiation; (C and D) Predictive models for micropapillary components in the training set (C) and in the validation set (D). Model 1: tumor size + gender; Model 2: Molde 1 + CEA; Model 3: Model 2 + Ki‐67 + tumor differentiations

Given the absence of independent external validation, we split patients into two data sets according to the operative date (training set: 2017–2019; validation set: 2020). Three models were constructed for the solid components: Model 1 (gender + tumor size), Model 2 (Model 1 + CEA), Model 3 (Model 2 + Ki‐67 + EGFR mutation + tumor differentiation). As shown in Figure 2A,B and Table 4, the model with only gender and tumor size yielded an AUC of 0.723 (training set) and 0.704 (validation set) for the solid component. Combination of CEA, gender, and tumor size led to a significant increase in the predictive accuracy (training set: 0.771, *p* = 0.009; validation set: 0.747, *p* = 0.034). Further, the integration of postoperative factors (.Ki‐67, EGFR mutation, and tumor differentiation) and Model 2 greatly improved the predictive accuracy (AUC: 0.962 in the training set and 0.942 in the validation set, *p* < 0.001).

**TABLE 4:** Comparison of AUC of predictive models for solid and micropapillary components

Models		Predictors	AUC			
			Training (2017–2019)	*p*	Validation (2020)	*p*
Solid component	Model 1	Gender + tumor size	0.723(0.687–0.762)	Ref.	0.704 (0.661–0.751)	Ref.
	Model 2	Model 1+ CEA	0.771(0.726–0.812)	**0.009**	0.747 (0.706–0.782)	**0.034**
	Model 3	Model 2 + Ki‐67 + EGFR + tumor differentiation	0.962(0.948–0.979)	**<0.001**	0.942 (0.908–0.968)	**<0.001**
Micropapillary component	Model 1	Gender + tumor size	0.699(0.636–0.760)	Ref.	0.711 (0.675–0.750)	Ref.
	Model 2	Model 1 + CEA	0.746(0.703–0.788)	**0.045**	0.753 (0.722–0.784)	**<0.001**
	Model 3	Model 2 + Ki‐67 + tumor differentiation	0.837(0.790–0.881)	**0.002**	0.901 (0.874–0.942)	**<0.001**

*P* value of <0.05 was marked in bold.

Likewise, we constructed three models (Model 1: gender + tumor size, Model 2: Model 1 + CEA, Model 3: Model 2 + Ki‐67 + tumor differentiation) to predict whether the micropapillary components existed in LUAD. The AUC of the model with only gender and tumor size was 0.699 and 0.711 in the training set and validation set, respectively (Table 4, Figure 2C,D). As expected, integration of CEA with gender and tumor size significantly improved the predictive performance with an AUC of 0.746 (training set, *p* = 0.045) and 0.753 (validation set, *p* < 0.001). When Ki‐67 and tumor differentiation were further integrated into Model 2, the predictive efficiencies had a great increase with an AUC of 0.837 in the training set and 0.901 in the validation set (*p* = 0.002 and *p* < 0.001, Figure 2C,D, Table 4).

## 4 DISCUSSION

Nowadays, more and more studies focus on the differential prognosis between LUAD histological subtypes. Patients with SPA or MPA might need a more conservative surgical procedure and show different responses to adjuvant therapies. Therefore, the early detection of SMC could have crucial impacts on the choice of surgical procedures and potential follow‐up adjuvant therapies.

CEA is a 180‐KDa glycoprotein normally expressed during fetal development but sharply declines before birth. Accordingly, serum CEA is usually less than 2.5 ng/ml in healthy adults but shows aberrantly increased expression in patients with malignant tumors.^21^, ^22^, ^23^ In this study, we found that the SPA had the highest serum CEA level, whereas the LPA harbored the lowest CEA level, which was consistent with previous studies.^18^, ^19^ Strikingly, the median levels of serum CEA in LPA, APA, and PPA were less than 2.5 ng/ml. However, patients with SPA or MPA had a median CEA level of more than 3.0 ng/ml. Consistently, LUAD samples with solid or micropapillary components also had a higher CEA level than those without solid or micropapillary components. The larger tumor size and stronger invasive ability of LUAD with solid or micropapillary components might account for the aberrant high level of serum CEA in SPA and MPA. As expected, CEA level was significantly associated with the tumor size, lymph node metastasis, and tumor differentiation.^24^ Besides, analyses in subgroup populations indicated that the expression levels of serum CEA in males and older patients were significantly higher than that in females and younger patients, respectively. Results from the multivariate regression analysis suggested that the impact of gender and age on the level of CEA might be independent of tumor size, histological subtypes, and tumor differentiation.

In addition to CEA, for the first time, we found that the CYFRA21‐1 level varied across histological subtypes of LUAD. The SPA had the highest level of CYFRA21‐1, whereas the LPA had the lowest CYFRA21‐1 level. Consistently, LUAD with solid or micropapillary components had a higher CYFRA21‐1 level than those without solid or micropapillary components. Similar to CEA, CYFRA21‐1 levels in males and older patients were significantly higher than those in females and younger patients, respectively. Besides, patients with larger tumor size, lymph node metastasis, and poor tumor differentiation harbored higher CYFRA21‐1 levels. Notably, the multivariate regression analysis showed that gender and tumor differentiation were not associated with the CYFRA21‐1 level, suggesting that the influence of gender and tumor differentiation on CYFRA21‐1 expression level might result from other underlying confounding factors.

Over the past few years, models for predicting SMC have been developed based on radiomics features and histopathological markers.^8^, ^9^, ^10^, ^11^, ^12^ In this study, we further evaluated the values of serum CEA and CYFRA21‐1 in the prediction of SMC in LUAD. The multivariate regression analysis indicated that the CYFRA21‐1 level was not independently associated with the SMC after adjusting for tumor size and other factors, while tumor size, gender, and CEA showed a significant association with the content of SMC. The integration of CEA with gender and tumor size significantly improved the predictive performance and achieved a moderate efficiency in predicting the SMC, comparable to models consisting of radiomics features.^10^, ^11^ Further, the inclusion of Ki‐67, EGFR mutation, and tumor differentiation greatly increased the predictive accuracy for solid components (0.962 in the training set and 0.942 in the validation set). Likewise, when CEA was integrated with gender and tumor size, the predictive ability for micropapillary components substantially increased. The combination of postoperative factors (Ki‐67 and tumor differentiation) and preoperative predictors further improved the predictive performance for the micropapillary components. The model consisting of CEA and other preoperative variables provided a practical tool to predict the SMC before the surgery, which could be of great significance in the choice of surgical procedures.^3^, ^6^ Models containing the postoperative clinicopathological characteristics, including Ki‐67 levels, EGFR mutation, and tumor differentiation, were valuable for patients who did not have a detailed pathological report. As far as we know, the LUAD subtype information is still not commonly reported in some hospitals (especially in the community hospitals). In addition, confirmation of the existence of SMC (≥5%) is subjective. Surgeons should pay attention when the predicted result is inconsistent with the pathological report.

Besides the great values in the early diagnosis and classification of lung cancer, CEA and CYFRA21‐1 correlated with the responses to chemotherapy and immunotherapy. For example, Wang et al. found that NSCLC patients with a higher CYFRA21‐1 level had a lower complete response rate of chemoradiotherapy compared with those with a low level of CYFRA21‐1 (2.9% vs. 20.3%).^25^ High levels of serum CEA and CYFRA21‐1 after two‐cycle adjuvant chemotherapy were associated with a poorer prognosis of NSCLC patients.^26^ CYFRA21‐1 integration with other predictors could predict the prognosis of advanced NSCLC patients treated with immunotherapy.^27^, ^28^, ^29^, ^30^ However, whether CEA and CYFRA21‐1 had different roles in the prognosis of LUAD histological subtypes need deeper survival analyses.

In summary, this study had three prominent advantages. First, this study had a large sample size with 3100 LUAD patients. Second, we used both the average values and grades of STM expression levels for the comparison. The independent effect of each factor on STM expression levels was further estimated using the regression analyses. Third, we integrated the STMs and other clinicopathological characteristics to predict the SMC in LUAD, preoperatively and postoperatively. However, this study also had some limitations. The underlying reasons for the differential CEA and CYFRA21‐1 levels among LUAD histological subtypes need in‐depth studies. In addition, 91.87% of patients enrolled in the current study were pathological stage Ι, only 8.13% of patients had stage ΙΙ or ΙΙΙ, which could restrict the applicability of our models.

## 5 CONCLUSIONS

In conclusion, serum CEA and CYFRA21‐1 levels differed across the predominant histological subtypes of LUAD. Serum CEA could be used as a valuable noninvasive predictor for the SMC in LUAD.

## CONFLICT OF INTEREST

Conflict of interest relevant to this article was not reported.

## AUTHOR CONTRIBUTIONS

Zhihua Li: Conceptualization, Writing‐original draft, Methodology, Formal analysis. Weibing Wu: Data curation, Methodology, Formal analysis, Resources, Funding acquisition. Xianglong Pan: Data curation, Writing‐original draft, Investigation. Fang Li: Resources, Data curation, Methodology, Supervision. Quan Zhu: Supervision, Resources, Data curation. Zhicheng He: Writing‐original draft, Data curation, Supervision, Resources. Liang Chen: Conceptualization, Writing‐original draft, Project administration, Resources, Funding acquisition.

## ETHICAL APPROVAL STATEMENT

This study was approved by the Ethical Committee of the First Affiliated Hospital of Nanjing Medical University and individual consent for this retrospective analysis was waived.

## Supporting information


Figure S1
Table S1–S7

## Data Availability

The data underlying this article cannot be shared publicly due to the privacy of individuals that participated in the study.

## References

[1] Zeng H , Chen W , Zheng R , et al. Changing cancer survival in China during 2003‐15: a pooled analysis of 17 population‐based cancer registries. Lancet Glob Health. 2018;6(5):e555‐e567. 29653628 10.1016/S2214-109X(18)30127-X

[2] Allemani C , Matsuda T , Di Carlo V , et al. Global surveillance of trends in cancer survival 2000‐14 (CONCORD‐3): analysis of individual records for 37 513 025 patients diagnosed with one of 18 cancers from 322 population‐based registries in 71 countries. Lancet. 2018;391(10125):1023‐1075. 29395269 10.1016/S0140-6736(17)33326-3 PMC5879496

[3] Nitadori J , Bograd AJ , Kadota K , et al. Impact of micropapillary histologic subtype in selecting limited resection vs lobectomy for lung adenocarcinoma of 2cm or smaller. J Natl Cancer Inst. 2013;105(16):1212‐1220. 23926067 10.1093/jnci/djt166 PMC3748005

[4] Ujiie H , Kadota K , Chaft JE , et al. Solid predominant histologic subtype in resected stage I lung adenocarcinoma is an independent predictor of early, extrathoracic, multisite recurrence and of poor postrecurrence survival. J Clin Oncol. 2015;33(26):2877‐2884. 26261257 10.1200/JCO.2015.60.9818 PMC4554749

[5] Zhao Y , Wang R , Shen X , et al. Minor components of micropapillary and solid subtypes in lung adenocarcinoma are predictors of lymph node metastasis and poor prognosis. Ann Surg Oncol. 2016;23(6):2099‐2105. 26842488 10.1245/s10434-015-5043-9 PMC4858562

[6] Su H , Xie H , Dai C , et al. Procedure‐specific prognostic impact of micropapillary subtype may guide resection strategy in small‐sized lung adenocarcinomas: a multicenter study. Ther Adv Med Oncol. 2020;12:1758835920937893. 32670422 10.1177/1758835920937893 PMC7336827

[7] Cha M J , Lee H Y , Lee K S , Jeong J Y , Han J , Shim Y M , et al. Micropapillary and solid subtypes of invasive lung adenocarcinoma: clinical predictors of histopathology and outcome, J Thorac Cardiovasc Surg. 147 (3) (2014) 921–928 e922. 24199757 10.1016/j.jtcvs.2013.09.045

[8] Song SH , Park H , Lee G , et al. Imaging phenotyping using radiomics to predict micropapillary pattern within lung adenocarcinoma. J Thorac Oncol. 2017;12(4):624‐632. 27923715 10.1016/j.jtho.2016.11.2230

[9] Yang SM , Chen LW , Wang HJ , et al. Extraction of radiomic values from lung adenocarcinoma with near‐pure subtypes in the International Association for the Study of Lung Cancer/the American Thoracic Society/the European Respiratory Society (IASLC/ATS/ERS) classification. Lung Cancer. 2018;119:56‐63. 29656753 10.1016/j.lungcan.2018.03.004

[10] Park S , Lee SM , Noh HN , et al. Differentiation of predominant subtypes of lung adenocarcinoma using a quantitative radiomics approach on CT. Eur Radiol. 2020;30(9):4883‐4892. 32300970 10.1007/s00330-020-06805-w

[11] He B , Song Y , Wang L , et al. A machine learning‐based prediction of the micropapillary/solid growth pattern in invasive lung adenocarcinoma with radiomics. Transl Lung Cancer Res. 2021;10(2):955‐964. 33718035 10.21037/tlcr-21-44 PMC7947386

[12] Zhao ZR , Lau RWH , Long H , Mok TSK , Chen GG , Underwood MJ , et al. Novel method for rapid identification of micropapillary or solid components in early‐stage lung adenocarcinoma, J Thorac Cardiovasc Surg. 2018;156(6):2310‐2318 e2312. 30180981 10.1016/j.jtcvs.2018.07.054

[13] Okamura K , Takayama K , Izumi M , Harada T , Furuyama K , Nakanishi Y . Diagnostic value of CEA and CYFRA 21‐1 tumor markers in primary lung cancer. Lung Cancer. 2013;80(1):45‐49. 23352032 10.1016/j.lungcan.2013.01.002

[14] Molina R , Marrades RM , Auge JM , et al. Assessment of a combined panel of six serum tumor markers for lung cancer. Am J Respir Crit Care Med. 2016;193(4):427‐437. 26465739 10.1164/rccm.201404-0603OC

[15] Zhang B , Niu X , Zhang Q , et al. Circulating tumor DNA detection is correlated to histologic types in patients with early‐stage non‐small‐cell lung cancer. Lung Cancer. 2019;134:108‐116. 31319968 10.1016/j.lungcan.2019.05.034

[16] Jiang ZF , Wang M , Xu JL . Thymidine kinase 1 combined with CEA, CYFRA21‐1 and NSE improved its diagnostic value for lung cancer. Life Sci. 2018;194:1‐6. 29247745 10.1016/j.lfs.2017.12.020

[17] Yu J , Du F , Yang L , et al. Identification of potential serum biomarkers for simultaneously classifying lung adenocarcinoma, squamous cell carcinoma and small cell carcinoma. Cancer Biomark. 2021;30(3):331‐342. 33361584 10.3233/CBM-201440 PMC12499969

[18] Lu F , Li S , Dong B , Zhang S , Lv C , Yang Y . Identification of lung adenocarcinoma mutation status based on histologic subtype: retrospective analysis of 269 patients. Thorac Cancer. 2016;7(1):17‐23. 26816535 10.1111/1759-7714.12265 PMC4718135

[19] Wang Z , Yang S , Lu H . Preoperative serum carcinoembryonic antigen levels are associated with histologic subtype, EGFR mutations, and ALK fusion in patients with completely resected lung adenocarcinoma. Onco Targets Ther. 2017;10:3345‐3351. 28744138 10.2147/OTT.S134452 PMC5511014

[20] Li Z , Li F , Pan C , et al. Tumor cell proliferation (Ki‐67) expression and its prognostic significance in histological subtypes of lung adenocarcinoma. Lung Cancer. 2021;154:69‐75. 33626488 10.1016/j.lungcan.2021.02.009

[21] Grunnet M , Sorensen JB . Carcinoembryonic antigen (CEA) as tumor marker in lung cancer. Lung Cancer. 2012;76(2):138‐143. 22153832 10.1016/j.lungcan.2011.11.012

[22] Yang G , Xiao Z , Tang C , Deng Y , Huang H , He Z . Recent advances in biosensor for detection of lung cancer biomarkers. Biosens Bioelectron. 2019;141:111416. 31279179 10.1016/j.bios.2019.111416

[23] Silsirivanit A . Glycosylation markers in cancer. Adv Clin Chem. 2019;89:189‐213. 30797469 10.1016/bs.acc.2018.12.005

[24] Tomita M , Ayabe T , Chosa E , Nakamura K . Correlation between serum carcinoembryonic antigen level and histologic subtype in resected lung adenocarcinoma. Asian Pac J Cancer Prev. 2015;16(9):3857‐3860. 25987049 10.7314/apjcp.2015.16.9.3857

[25] Wang J , Yi Y , Li B , et al. CYFRA21‐1 can predict the sensitivity to chemoradiotherapy of non‐small‐cell lung carcinoma. Biomarkers. 2010;15(7):594‐601. 20649505 10.3109/1354750X.2010.504308

[26] Lin XF , Wang XD , Sun DQ , Li Z , Bai Y . High serum CEA and CYFRA21‐1 levels after a two‐cycle adjuvant chemotherapy for NSCLC: possible poor prognostic factors. Cancer Biol Med. 2012;9(4):270‐273. 23691489 10.7497/j.issn.2095-3941.2012.04.009 PMC3643679

[27] Chai R , Fan Y , Zhao J , He F , Li J , Han Y . Prognostic nomogram on clinicopathologic features and serum indicators for advanced non‐small cell lung cancer patients treated with anti‐PD‐1 inhibitors. Ann Transl Med. 2020;8(17):1078. 33145297 10.21037/atm-20-4297 PMC7575979

[28] Zhang Z , Yuan F , Chen R , et al. Dynamics of serum tumor markers can serve as a prognostic biomarker for Chinese advanced non‐small cell lung cancer patients treated with immune checkpoint inhibitors. Front Immunol. 2020;11:1173. 32587591 10.3389/fimmu.2020.01173 PMC7298878

[29] Dal Bello MG , Filiberti RA , Alama A , et al. The role of CEA, CYFRA21‐1 and NSE in monitoring tumor response to nivolumab in advanced non‐small cell lung cancer (NSCLC) patients. J Transl Med. 2019;17(1):74. 30849967 10.1186/s12967-019-1828-0 PMC6408784

[30] Dall'olio FG , Abbati F , Facchinetti F , et al. CEA and CYFRA 21‐1 as prognostic biomarker and as a tool for treatment monitoring in ?>x..X .,advanced NSCLC treated with immune checkpoint inhibitors. Ther Adv Med Oncol. 2020;12:1758835920952994. 33193825 10.1177/1758835920952994 PMC7607728

