# Supplementary material for: Serum tumor markers level and their predictive values for solid and micropapillary components in lung adenocarcinoma
Source: Cancer Med. 2022 Mar 14;11(14):2855–64. doi: 10.1002/cam4.4645 (PMC9302275; doi:10.1002/cam4.4645)
Supplement: Supplementary file 1 — Figure S1 Table S1–S7 [file CAM4-11-2855-s001.docx]

**Supplementary Table 1. Characteristics of study subjects in this study.**

| **Characteristics** | **N** | **%** |
| --- | --- | --- |
| **Gender** |  |  |
| Male | 1227 | 39.58% |
| Female | 1873 | 60.42% |
| **Age (Mean±SD)** | 59.22±10.61 |  |
| < 60 | 1480 | 47.74% |
| ≥ 60 | 1620 | 52.26% |
| **Tumor location** |  |  |
| Right upper | 1048 | 33.81% |
| Right middle | 251 | 8.10% |
| Right lower | 554 | 17.87% |
| Left upper | 781 | 25.19% |
| Left lower | 466 | 15.03% |
| **Tumor size (mm)** | 17.11±8.69 |  |
| < 10 | 729 | 23.52% |
| 10~20 | 1637 | 52.81% |
| 20~30 | 546 | 17.61% |
| ≥ 30 | 188 | 6.06% |
| **LN status** |  |  |
| N0 | 2877 | 92.81% |
| N+ | 223 | 7.19% |
| **Pathologic Stage** |  |  |
| I | 2848 | 91.87% |
| II | 111 | 3.58% |
| III | 141 | 4.55% |
| **Histological subtypes** |  |  |
| LPA | 931 | 30.03% |
| APA | 1933 | 62.35% |
| PPA | 135 | 4.35% |
| SPA | 80 | 2.58% |
| MPA | 21 | 0.68% |
| **Tumor Differentiation** | |  |
| I | 562 | 18.13% |
| II | 569 | 18.35% |
| III | 403 | 13.00% |
| NA | 1566 | 50.52% |
| **EGFR mutation** |  |  |
| With | 1076 | 70.79% |
| Without | 444 | 29.21% |
| NA | 1580 | / |
| **AFP** |  |  |
| Median(IQR) | 2.74(1.96-3.90) |  |
| Positive | 5 | 0.16% |
| **CEA** |  |  |
| Median(IQR) | 2.03(1.32-3.13) |  |
| Positive | 350 | 11.29% |
| **CA199** |  |  |
| Median(IQR) | 10.16(6.70-15.72) |  |
| Positive | 55 | 1.78% |
| **CA724** |  |  |
| Median(IQR) | 1.75(1.08-3.84) |  |
| Positive | 337 | 10.89% |
| **CYFRA21-1** |  |  |
| Median(IQR) | 2.06(1.56-2.69) |  |
| Positive | 393 | 12.68% |
| **NSE** |  |  |
| Median(IQR) | 16.67(14.24-20.07) |  |
| Positive | 1659 | 53.52% |

**NA: not available; IQR: interquartile range;**

**Supplementary Table 2. Expression levels of STMs in male and female LUAD patients.**

| **Characteristics** | Males (n=1227) | Females (n=1873) | ***P*** |
| --- | --- | --- | --- |
| **Tumor size (cm)** | 1.81±0.95 | 1.65±0.81 | **<0.001** |
| **Ki-67** | 15.00(7.00-30.00) | 10.00(5.00-20.00) | **<0.001** |
| **AFP** | 2.81(2.10-3.92) | 2.67(1.88-3.88) | **0.006** |
| **CEA** | 2.38(1.55-3.65) | 1.83(1.21-2.80) | **<0.001** |
| **CA199** | 10.17(6.53-15.52) | 10.16(6.76-15.82) | 0.458 |
| **CA724** | 1.75(1.12-3.69) | 1.78(1.05-3.90) | 0.777 |
| **CYFRA21-1** | 2.11(1.63-2.79) | 2.01(1.50-2.62) | **<0.001** |
| **NSE** | 17.40(14.64-20.99) | 16.28(14.03-19.42) | **<0.001** |
| **AFP_positive(%)^*^** | 3 (0.2%) | 2(0.1%) | 0.633 |
| **CEA_positive(%)^*^** | 198(16.1%) | 152(8.1%) | **<0.001** |
| **CA199_positive(%)^*^** | 25(2.0%) | 30(1.6%) | 0.450 |
| **CA724_positive(%)^*^** | 111(9.1%) | 226(12.1%) | **0.010** |
| **CYFRA21-1_positive(%)^*^** | 175(14.3%) | 218(11.6%) | **0.036** |
| **NSE_positive(%)^*^** | 726(59.3%) | 933(49.8%) | **<0.001** |

**^*^Patients with abnormal levels of STMs.**

**Supplementary Table 3. Expression levels of STMs in older and younger LUAD patients.**

| **Characteristics** | age < 60 (n=1480) | Age ≥ 60 (n=1620) | ***P*** |
| --- | --- | --- | --- |
| **Tumor size (cm)** | 1.55±0.86 | 1.86±0.85 | **<0.001** |
| **Ki-67** | 10.00(5.00-20.00) | 10.00(5.25-30.00) | **<0.001** |
| **AFP** | 2.70(1.90-3.98) | 2.77(2.04-3.86) | 0.326 |
| **CEA** | 1.67(1.11-2.56) | 2.39(1.58-3.55) | **<0.001** |
| **CA199** | 9.46(6.27-14.27) | 10.85(7.25-17.03) | **<0.001** |
| **CA724** | 1.75(1.09-3.81) | 1.75(1.08-3.84) | 0.998 |
| **CYFRA21-1** | 1.88(1.40-2.44) | 2.22(1.72-2.91) | **<0.001** |
| **NSE** | 16.59(14.04-20.35) | 16.69(14.38-19.88) | 0.524 |
| **AFP_positive(%)** | 3(0.2%) | 2(0.1%) | 0.921 |
| **CEA_positive(%)** | 111(7.5%) | 239(14.8%) | **<0.001** |
| **CA199_positive(%)** | 21(1.4%) | 34(2.1%) | 0.191 |
| **CA724_positive(%)** | 171(11.6%) | 166(10.3%) | 0.283 |
| **CYFRA21-1_positive(%)** | 125(8.4%) | 268(16.6%) | **<0.001** |
| **NSE_positive(%)** | 775(52.4%) | 884(54.7%) | 0.212 |

**Supplementary Table 4. STMs levels according to tumor size.**

| **Characteristics** | **≤ 10 mm** | **10-20 mm** | **20-30 mm** | **>30 mm** | ***P*** |
| --- | --- | --- | --- | --- | --- |
|  | **n=729** | **n=1637** | **n=546** | **n=188** |  |
| **Ki-67** | 5.00(5.00-10.00) | 10.00(5.00-20.00) | 20.00(10.00-35.00) | 30.00(15.00-50.00) | **<0.001** |
| **AFP** | 2.64(1.90-3.86) | 2.76(1.95-3.94) | 2.79(2.05-3.81) | 2.80(2.05-3.89) | 0.616 |
| **CEA** | 1.59(1.08-2.41) | 1.95(1.31-2.87) | 2.77(1.82-4.28) | 3.39(2.18-7.61) | **<0.001** |
| **CA199** | 9.91(6.53-14.39) | 10.09(6.70-15.67) | 10.61(6.89-16.86) | 11.68(7.39-20.35) | **0.004** |
| **CA724** | 1.78(1.11-3.85) | 1.73(1.08-3.69) | 1.87(1.09-4.33) | 1.74(1.03-3.45) | 0.632 |
| **CYFRA21-1** | 1.92(1.44-2.46) | 2.05(1.54-2.68) | 2.16(1.64-2.77) | 2.46(1.94-3.47) | **<0.001** |
| **NSE** | 16.90(14.22-20.23) | 16.61(14.25-20.20) | 16.44(14.16-19.62) | 16.94(14.53-20.35) | 0.398 |
| **AFP_positive(%)** | 0(0.0) | 2(0.1%) | 2(0.4%) | 1(0.5%) | 0.225 |
| **CEA_positive(%)** | 28(3.8%) | 130(7.9%) | 124(22.7%) | 68(36.2%) | **<0.001** |
| **CA199_positive(%)** | 5(0.7%) | 22(1.3%) | 16(2.9%) | 12(6.4%) | **<0.001** |
| **CA724_positive(%)** | 75(10.3%) | 181(11.1%) | 61(11.2%) | 20(10.6%) | 0.944 |
| **CYFRA21-1_positive(%)** | 51(7.0%) | 217(13.3%) | 71(13.0%) | 54(28.7%) | **<0.001** |
| **NSE_positive(%)** | 404(55.4%) | 866(52.9%) | 281(51.7%) | 108(57.4%) | 0.361 |

**Supplementary Table 5. STMs levels in LUAD patients with and without lymph node metastasis.**

| **Characteristics** | **Lymph node metastasis** | | ***P*** |
| --- | --- | --- | --- |
|  | **N0(n=2877)** | **N+(n=223)** |  |
| **Tumor size (cm)** | 1.62±0.77 | 2.81±1.26 | **<0.001** |
| **Ki-67** | 10.00(5.00-20.00) | 40.00(25.00-50.00) | **<0.001** |
| **AFP** | 2.74(1.96-3.88) | 2.74(1.97-4.32) | 0.688 |
| **CEA** | 1.96(1.29-2.99) | 3.53(2.12-8.11) | **<0.001** |
| **CA199** | 10.10(6.71-15.53) | 11.34(6.15-18.41) | 0.163 |
| **CA724** | 1.77(1.09-3.83) | 1.67(1.06-4.33) | 0.980 |
| **CYFRA21-1** | 2.04(1.55-2.66) | 2.30(1.71-3.31) | **<0.001** |
| **NSE** | 16.66(14.24-20.04) | 16.78(14.31-20.49) | 0.587 |
| **AFP_positive(%)** | 2(0.1%) | 3(1.3%) | **<0.001** |
| **CEA_positive(%)** | 255(8.9%) | 95(41.9%) | **<0.001** |
| **CA199_positive(%)** | 45(1.6%) | 10(4.4%) | **0.004** |
| **CA724_positive(%)** | 308(10.7%) | 29(12.9%) | 0.377 |
| **CYFRA21-1_positive(%)** | 335(11.7%) | 58(25.7%) | **<0.001** |
| **NSE_positive(%)** | 1536(53.5%) | 123(54.4%) | 0.842 |

**Supplementary Table 6. STMs levels in LUAD patients according to tumor differentiation.**

| **Characteristics** | ΙΙΙ (n=403) | ΙΙ (n=569) | Ι (n=562) | *P* |
| --- | --- | --- | --- | --- |
| **Gender = female(%)** | 185(45.9%) | 361(63.4%) | 358(63.7%) | **<0.001** |
| **Age** | 60.43±9.96 | 60.20±9.90 | 58.60±10.75 | **0.007** |
| **Tumor size (cm)** | 2.44±1.11 | 1.76±0.75 | 1.35±0.56 | **<0.001** |
| **Ki-67** | 30.00(20.00-50.00) | 10.00(5.00-20.00) | 5.00(5.00-10.00) | **<0.001** |
| **AFP** | 2.77(2.07-4.16) | 2.87(2.03-3.90) | 2.59(1.90-3.80) | 0.097 |
| **CEA** | 2.68(1.77-4.88) | 2.00(1.34-3.03) | 1.81(1.22-2.74) | **<0.001** |
| **CA199** | 10.67(6.37-17.05) | 10.14(7.10-15.49) | 9.88(6.56-14.63) | 0.311 |
| **CA724** | 1.85(1.06-4.09) | 1.87(1.07-3.98) | 1.79(1.06-3.77) | 0.833 |
| **CYFRA21-1** | 2.18(1.64-2.86) | 2.04(1.53-2.62) | 1.97(1.49-2.64) | **<0.001** |
| **NSE** | 16.46(13.99-19.81) | 16.30(13.89-19.83) | 16.80(14.37-20.02) | 0.324 |
| **AFP_positive(%)** | 1(0.2%) | 0 | 2(0.4%) | 0.384 |
| **CEA_positive(%)** | 106(26.3%) | 49(8.6%) | 29(5.2%) | **<0.001** |
| **CA199_positive(%)** | 17(4.2%) | 12(2.1%) | 4(0.7%) | **0.001** |
| **CA724_positive(%)** | 52(12.9%) | 71(12.5%) | 57(10.2%) | 0.324 |
| **CYFRA21-1_positive(%)** | 72(17.9%) | 73(12.9%) | 62(11.0%) | **0.008** |
| **NSE_positive(%)** | 205(51.0%) | 285(50.1%) | 308(54.8%) | 0.251 |

**Supplementary Table 7. Univariate and multivariate regression analyses of CEA and CYFRA21-1 abnormal levels in LUAD patients.**

| **Characteristics** | **CEA** | | |  | **CYFRA21-1** | | |
| --- | --- | --- | --- | --- | --- | --- | --- |
|  | **Univariate** | **Multivariate** | ***P*** |  | **Univariate** | **Multivariate** | ***P*** |
| Gender (Female) | 0.46(0.37-0.57) | 0.52(0.41-0.65) | **<0.001** |  | 0.79(0.64-0.98) | 0.90(0.72-1.12) | 0.327 |
| Age | 1.04(1.03-1.05) | 1.03(1.01-1.04) | **<0.001** |  | 1.05(1.04-1.06) | 1.04(1.03-1.05) | **<0.001** |
| Size |  |  | **<0.001** |  |  |  | **<0.001** |
| ≤ 1cm | Ref | Ref | / |  | Ref | Ref | / |
| 1~2 cm | 2.16(1.42-3.28) | 1.65(1.07-2.53) | 0.023 |  | 2.03(1.48-2.8) | 1.56(1.12-2.16) | 0.008 |
| 2~3 cm | 7.36(4.80-11.28) | 5.01(3.20-7.84) | <0.001 |  | 1.99(1.36-2.91) | 1.35(0.90-2.01) | 0.143 |
| > 3 cm | 14.19(8.77-22.94) | 8.39(5.04-13.95) | <0.001 |  | 5.36(3.50-8.20) | 3.49(2.22-5.49) | <0.001 |
| Histological subtypes |  |  | **0.007** |  |  |  | 0.195 |
| LPA | Ref | Ref | / |  | Ref | Ref | / |
| APA | 2.37(1.74-3.21) | 1.54(1.11-2.12) | 0.009 |  | 1.43(1.11-1.84) | 1.21(0.93-1.58) | 0.149 |
| PPA | 2.18(1.21-3.94) | 1.50(0.81-2.79) | 0.199 |  | 1.40(0.82-2.41) | 1.23(0.71-2.15) | 0.459 |
| SPA | 8.27(4.83-14.18) | 2.64(1.46-4.77) | 0.001 |  | 2.89(1.65-5.05) | 1.86(1.02-3.39) | **0.043** |
| MPA | 8.12(3.15-20.95) | 3.16(1.13-8.88) | 0.029 |  | 0.96(0.22-4.19) | 0.54(0.12-2.42) | 0.418 |
| EGFR1: 1 vs 0 | 0.54(0.38-0.75) | 0.64(0.44-0.94) | **0.022** |  | 0.97(0.68-1.40) | 1.02(0.69-1.51) | 0.931 |
| Differentiation |  |  | **<0.001** |  |  |  | 0.478 |
| ΙΙΙ | Ref | Ref | / |  | Ref | Ref | / |
| ΙΙ | 0.26(0.18-0.38) | 0.40(0.26-0.60) | <0.001 |  | 0.68(0.48-0.97) | 0.78(0.53-1.17) | 0.232 |
| Ι | 0.15(0.10-0.24) | 0.20(0.10-0.38) | <0.001 |  | 0.57(0.39-0.82) | 0.80(0.48-1.33) | 0.391 |

LPA: lepidic predominant adenocarcinoma; APA: acinar predominant adenocarcinoma; PPA: papillary predominant adenocarcinoma; MPA: micropapillary predominant adenocarcinoma; SPA: solid predominant adenocarcinoma.


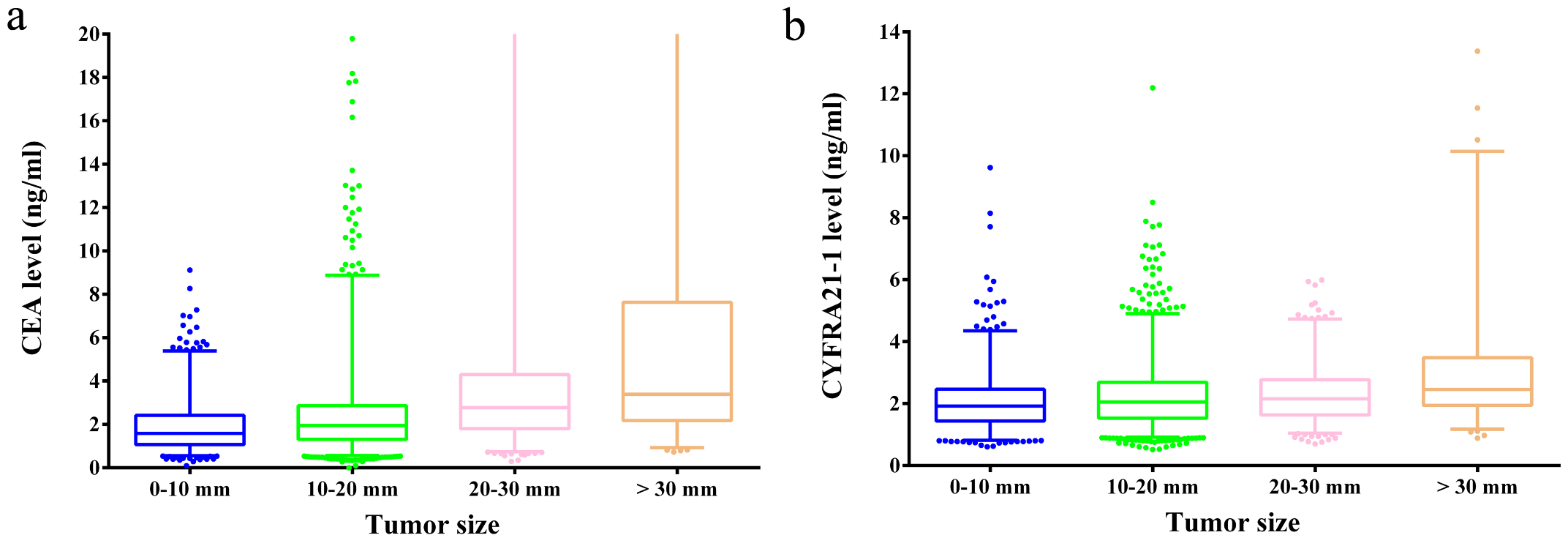


**Supplementary Figure 1. Serum CEA and CYFRA21-1 levels increased with the increase of tumor size.**


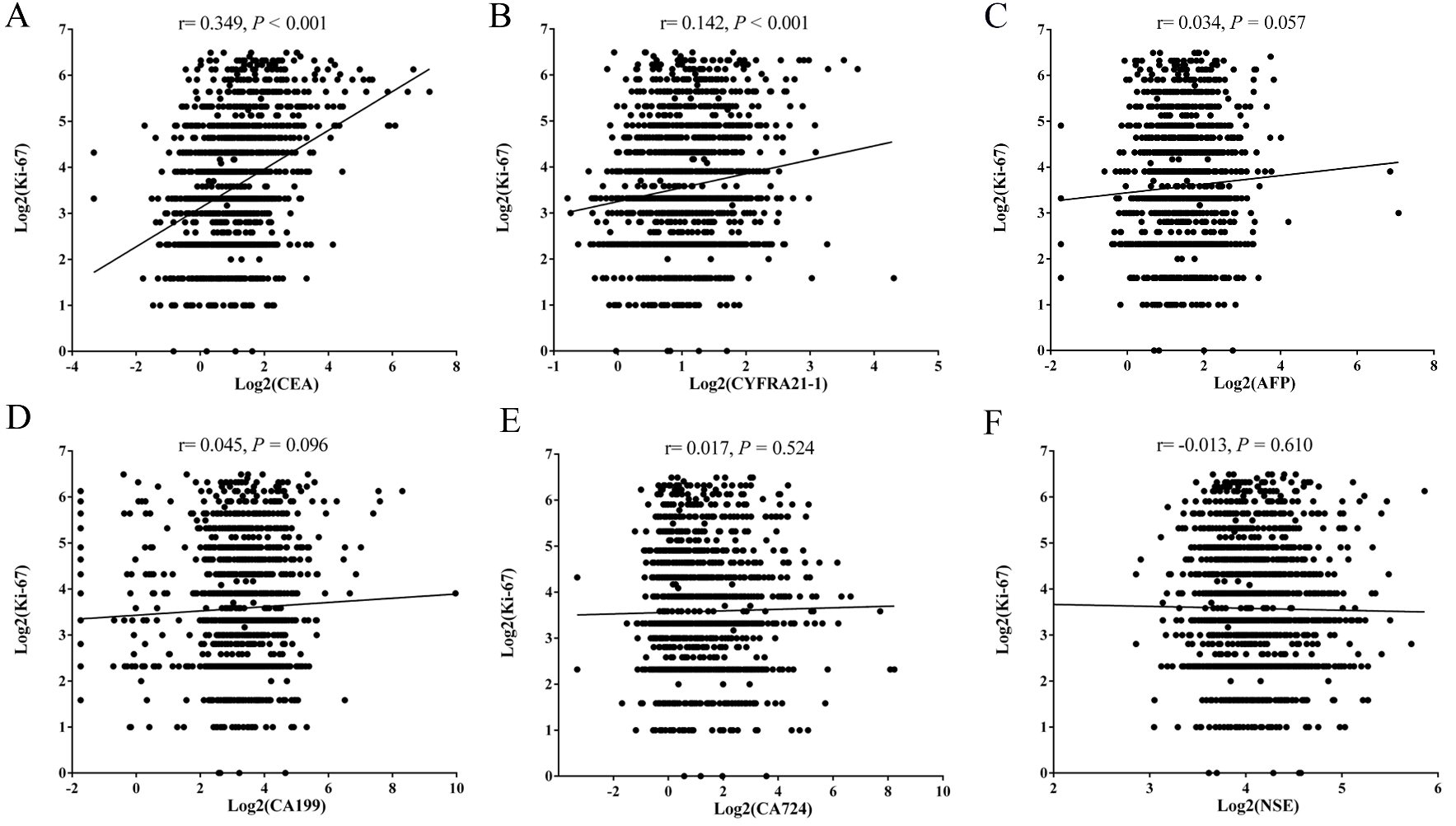


**Supplementary Figure 2. Correlation between STMs levels and Ki-67 expression levels in LUAD tissues.**

Serum CEA (a) and CYFRA21-1 (b) were significantly correlated with the expression level of Ki-67 in LUAD tissues, while AFP (c), CA199 (d), CA724 (e) and NSE (f) showed no significant correlation with Ki-67.
